# Presentation and Management of Neglected Developmental Dysplasia of Hip (DDH): 8-years’ experience with single stage triple procedure at National Institute of Rehabilitation Medicine, Islamabad, Pakistan

**DOI:** 10.12669/pjms.343.14392

**Published:** 2018

**Authors:** Farid Ullah Khan Zimri, Syed Shujaat Ali Shah, Muhammad Saaiq, Faisal Qayyum, Muhammad Ayaz

**Affiliations:** 1 Dr. Farid Ullah Khan Zimri, FCPS. Department of Orthopedic Surgery, National Institute of Rehabilitation Medicine (NIRM), Islamabad, Pakistan; 2 Dr. Syed Shujaat Ali Shah, MS. Department of Orthopedic Surgery, National Institute of Rehabilitation Medicine (NIRM), Islamabad, Pakistan; 3 Dr. Muhammad Saaiq, FCPS. Department of Orthopedic Surgery, National Institute of Rehabilitation Medicine (NIRM), Islamabad, Pakistan; 4 Dr. Faisal Qayyum, MBBS. Department of Orthopedic Surgery, National Institute of Rehabilitation Medicine (NIRM), Islamabad, Pakistan; 5 Dr. Muhammad Ayaz, MBBS. Department of Orthopedic Surgery, National Institute of Rehabilitation Medicine (NIRM), Islamabad, Pakistan

**Keywords:** Developmental dysplasia of hip, Triple procedure, Femoral shortening, Salter’s osteotomy

## Abstract

**Objective:**

To document the clinical presentation of neglected DDH and evaluate the outcome of triple procedure.

**Methods:**

It was a descriptive case series study, conducted at the Department of Orthopedic Surgery, National Institute of Rehabilitation Medicine (NIRM), Islamabad over a period of 8-years. It included children aged >1 and <9 years who underwent the triple procedure of open reduction, femoral shortening and Salter’s osteotomy. Clinical evaluation was performed using McKay’s criteria. Tonnis classification and Severin’s scoring system were employed for the radiological evaluation.

**Results:**

There were 193 children with 213 DDH affected hips. The mean age was 3.31±1.6 years. The preoperative severity of the femoral head dislocation per Tonnis classification was Grade-I in 7.98%(n=17), Grade-II in 48.35%(n=103) and Grade-III in 43.66%(n=93) hips. The postoperative MacKay criteria was ’Good’ to ’Excellent’ in 193(90.61%) hips. The postoperative Severin’s class was I in 113(53%) hips, II in 48(22.53%) hips, III in 43(20.18%) and IV in 9(4.22%) hips. The preoperative acetabular index ranged from 39° to 51° with a mean of 43.91±3.69°. The mean postoperative AI was 18.42±2.99°. The postoperative centre edge angle ranged from 21° to 26° with a mean 23.18 ±1.35°.

**Conclusions:**

The single stage triple procedure offers the surgical remedy of choice with favourable results for managing neglected and late diagnosed DDH among children aged 1-8 years.

## INTRODUCTION

Developmental dysplasia of hip (DDH) represents a spectrum of anatomic hip abnormalities that affect the development and stability of the hip during the critical growth period. Theses abnormalities range from subtle acetabular dysplasia to the more sinister and complete subluxation or dislocation of the femoral head.^1^-^3^ The incidence of DDH varies considerably in different parts of the world and range between 0.5% and 1.5%. The underlying causes are multi-factorial and include genetic and mechanical factors. The genetic factors are indicated by the increased incidence in female children and those with a positive family history. The mechanical factors are reflected by the increased risk among children born with breech presentation, advanced maternal age, postmaturity, oligohydramnios, or any other crowding intrauterine conditions.^4^-^6^

The goal of treatment is to achieve a stable concentric reduction without disrupting the critical circulation of femoral head and to maintain this reduction during childhood and adolescence. Early reduction accelerates normal development of both the femoral head and acetabulum at the hip joint. Open reduction is indicated when closed concentric reduction cannot be achieved satisfactorily. For instance, in older children adequate reduction is a formidable foe when the femoral head is flattened or when the acetabulum is dysplastic. Reduction is also hindered by the associated soft tissue contractures around the joint. Failure to treat the condition at an early age leads to gait abnormalities, limitation of hip motion, joint pain and disabling progressive osteoarthritis at an early age.^1^-^3^,^7^

The present study was carried out to document the clinical presentation of neglected DDH and evaluate the outcome of triple procedure in terms of clinical and radiologic criteria.

## METHODS

This descriptive case series study was carried out at the Department of Orthopedic Surgery, National Institute of Rehabilitation Medicine (NIRM), Islamabad over an 8-years period from January 1, 2010 to December 7, 2017.

All children of either gender aged >1 and <9 years who presented with neglected or late diagnosed DDH were included in the study. Exclusion criteria were children <1 year, neuromuscular disorders, teratogenic hips, arthrogryposis multiplex congenital, those who previously received any form of DDH treatment elsewhere and all cases where triple procedure was contraindicated. All the children were hospitalized. Informed consent was taken from the parents. The study was approved by the hospital ethics committee.

Thorough clinical evaluation was made including standard pelvic X-rays of anteroposterior and lateral views. Modified McKay’s criteria^8^ were employed for expressing the preoperative clinical presentation of the patients as well as their postoperative clinical condition. (Table-I).

**Table-I:** McKay’s clinical criteria.

Grade	Rating	Description
I	Excellent	Stable painless hip; Negative Trendelenberg sign; Full range of motion (ROM)
II	Good	Slight limp; Slight reduction in ROM
III	Fair	Positive Trendelenberg sign; Moderate limp; Limited ROM
IV	Poor	Positive Trendelenberg sign. Unstable and/or Painful hip;

Preoperative severity of the femoral head dislocation was recorded by employing the Tonnis classification.^9^ (i.e. Grade-I: The ossification centre of the femoral head is displaced laterally but still inferior to the superolateral corner of the true acetabulum, Grade-II: The ossification centre is at the level of the superolateral corner of the true acetabulum and Grade- III: The ossification centre is superior to the superolateral margin of the true acetabulum). The postoperative radiographic results were documented by employing the Severin’s classification.^10^ (Table-II) On preoperative X-rays, the acetabular index (AI) and on postoperative X- rays, the center edge angle (CEA) and AI were measured.

**Table-II:** Severin’s radiographic classification for postoperative outcome.

Class/ Grade	Description	Centre-Edge Angle (Degrees)
I	Normal appearance	≥15(5-13 Years) ≥20(>14 Years)
II	Mild deformity of femoral head, neck or acetabulum	≥15(5-13 Years) ≥20(>14Years)
III	Dysplasia/ moderate deformity	<15(5-13 Years) <20(>14 Years)
IV	Subluxation of femoral head	-
V	Articulation of femoral head with false acetabulum	-
VI	Redislocation	-

All the children underwent single-stage triple procedure under general anesthesia. Children with bilateral involvement were operated in two different sessions, six months apart. Adductor tenotomy was performed at the outset of the operation. Anterior approach via bikini incision was employed. The lateral cutaneous nerve of thigh was safeguarded. The flat femoral nerve and vessels were located and safeguarded as the medial limit of dissection. The muscles were reflected from the inner and outer aspects of the iliac crest to expose the hip joint. The iliopsoas muscle was lengthened by preserving the bulky belly while performing recession tenotomy of its deep lying tendon. Distal to the anterior inferior iliac spine, the direct and oblique heads of the rectus femoris were tenotomized. Next the abductor muscles were separated from the hip joint capsule. The capsule was opened through a linear incision oriented along the acetabular edge. Digit was inserted to verify and dissect off the ligamentum teres, pulvinar and the transverse acetabular ligament. Next gentle traction was applied to assess reducibility of the femoral head to the true acetabulum without pressure. For femoral shortening a separate lateral approach was used. The proximal end of femur was exposed subperiosteally and a transverse osteotomy was performed at a level distal to the lesser trochanter. The amount of overlap at osteotomy site was resected from the femoral shaft using an oscillating saw. Osteosynthesis was performed using a 6-7 hole dynamic compression plate. The femoral shortening allowed tension free stable concentric reduction of the femoral head. Once reduced concentrically, the stability of the hip joint was assessed with the limb in the weight-bearing position. (i.e. in slight abduction and extension). Derotation was achieved by externally rotating the distal femoral fragment in relation to the proximal fragment until the patella pointed directly forward before applying the plate. Salter’s pelvic osteotomy and capsulorrhaphy were performed in a standard fashion. The former was performed where the acetabular index was >40°.

Postoperatively the hips were immobilized with a double hip spica cast. The hip joints were placed in 90°-100° of flexion and 40°-55° of abduction with neutral internal rotation. The spica was continued for three months. Home physiotherapy was initiated after removal of the spica. The first spica cast was changed after four weeks postoperatively with removal of the hip joint K-wire. The osteotomy site K-wire was removed after X-ray confirmation of the consolidation at the osteotomy site usually three months postoperatively.

The patients were then regularly followed up in the outpatient department on monthly basis for the first 3 months and then every three monthly till the end of one year. The development of the operated hip joint was monitored through serial X-rays. The pelvis anteroposterior views were evaluated for the Tonnis grading of the femoral head displacement, measuring the acetabular index and CEA, the continuity of the Shenton’s line and any evidence of AVN.

**Fig.1:**
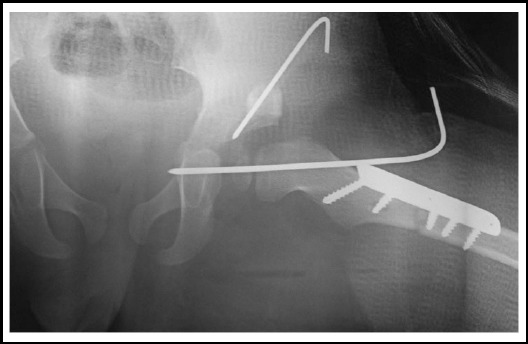
Radiographic illustration of the triple procedure.

The outcome measure of triple procedure was treatment success which was evaluated clinically using McKay’s criteria and radiologically using Severin’s criteria. The final results of these were documented at one year follow up. The data were analysed through SPSS-17 to measure the outcomes. Fig.1 is the radiographic illustration of the triple procedure.

## RESULTS

We had a total of 193 children in our study with 213 DDH affected hips. There were 152 females (78.75%) while 41 males (21.24%). Children with left sided DDH were 127(59.62%), right sided 46 (21.59%) and bilateral DDH were 20(9.38%).

The age ranged from 1-8 years with a mean of 3.31±1.6 years. The hospital stay was 7-15 days with a mean stay of-8.03±1.98 days.

Results of clinical evaluation as assessed according to the McKay’s criteria are as follows: Preoperatively, Grade-III in 115(53.99%) while Grade-IV in 98(46%) hips. Postoperatively, Grade-I in 89(41.78%), Grade-II in 104(48.82%), Grade-III in 13(6.10%) and Grade-IV in 7(3.28%) hips.

The preoperative severity of the femoral head dislocation per Tonnis classification was as follows: Grade I in 7.98%(n=17), Grade-II in 48.35%(n=103) and Grade-III in 43.66%(n=93) hips.

All hips had an acetabular index(AI) of over 30 degrees preoperatively. The preoperative AI ranged from 39° to 51° with a mean of 43.91±3.69°. The postoperative AI was 15° -25° with a mean of 18.42±2.99°. Preoperatively CEA was negative among all cases whereas the postoperative CEA ranged from 21° to 26° with a mean of 23.18±1.35°.

The radiographic outcome as per Severin’s classification is as follows: Preoperatively, Grade-IV in 110(51.64%) and grade V in 103(48.35%) hips. Postoperatively, Grade-I in 113(53%), Grade-II in 48(22.53%), Grade-III in 43(20.18%), and Grade-IV in 9(4.22%) hips.

Among our share of complications included AVN and re-dislocation three cases each (1.40%). Moderate stiffness following removal of hip spica was observed among 13 cases (6.10%).

## DISCUSSION

We found high frequency of neglected DDH in our patients. Our centre is located in the capital city of Pakistan and severs as a referral centre for the rehabilitation of disabled children from all over the country. The Orthopedic unit has one hundred beds with a 15 bedded bay dedicated for DDH children We receive patients particularly from areas such as Gilgit-Baltistan, Khyber-Pakhtunkhwa, Azad Jammu Kashmir, Federally administered tribal areas, Afghanistan, upper Punjab in addition to the main catchment areas of the twin cities of Islamabad and Rawalpindi. In our country, we neither have a screening programme for timely detection of DDH, nor have any structured referral system for these children. Hence majority of our children present with neglected DDH or late diagnosed DDH and need more aggressive surgical remedies such as the triple procedure.

We observed relatively more frequent affliction of females than males. Left side was also more frequently affected than the right sided hip. Our current findings conform to most of the reported literature.^1^-^3^

In our study we employed the single-stage triple surgical procedure. This extensive procedure consists of open reduction, femoral shortening, and Salter’s pelvic osteotomy. It is technically more demanding procedure than a staged procedure. Our results favourably compare with most of the published literature. Vallamshetla et al.^11^ in their series reported 100% good or excellent clinical results using McKay’s criteria while 100% Severin’s classification class I and II. Similarly Umer et al.^12^ have reported 86.2% good to excellent clinical results and 51.7% Severin’s class I in their series.

Open surgical reduction is the mainstay of the triple procedure Indeed it is the most effective modality for reducing DDH in grown up children. Certainly reduction in an older child often poses more challenge owing to a host of reasons. For instance, there is associated adaptive shortening of the soft tissues, capsular constriction, increased femoral anteversion, acetabular dysplasia, presence of obstructing structures such as the fibrofatty tissue in the acetabulum, hypertrophied ligamentum teres, the transverse acetabular ligament and fixed inversion of limbus.^1^-^4^,^13^,^14^ Femoral diaphyseal shortening is superior to traction as an aid in operative reduction of DDH in older children. It greatly decreases the rate of AVN and also decreases the risk of redislocation.^3^,^15^-^17^ The femoral shortening is particularly helpful in high dislocations to prevent undue pressure on the femoral head, which is the main cause of postoperative AVN, joint stiffness and reduction failures.

We employed Salter osteotomy in our patients and found it to be easy and useful for stabilizing hips after open reduction. It improves the cover of the femoral head and provides stability in the weight-bearing position. It can be done safely and reliably without any increase in the risk of AVN. Several other types of pelvic osteotomies have also been described to stabilize the reduced hip in older children.^17^-^19^

In our study we set the upper age limit of eight years in unilateral while six years in bilateral cases of DDH to be managed with the standard triple procedure. Although most of the authorities agree to eight years as the upper age limit for this treatment, Ok IY et al.^20^ have recommended that, if there is a high likelihood of achieving a functionally good hip joint with biological remodeling, an open reduction is a reasonable strategy for an untreated dislocation in patients even older than eight years of age. They found that joint remodeling continued even after this age after a concentric reduction of the DDH.

## CONCLUSION

The single stage triple procedure entailing open reduction, femoral shortening and Salter’s osteotomy offers the surgical remedy of choice with favourable and lasting results for managing neglected DDH among children aged 1-8 years. Attention to fine details regarding correction of the complex morbid anatomy holds the key to successful outcome.

### Authors’ Contributions

FUKZ and MS designed the study and wrote the manuscript.

SSAS, FQ and MA collected data and analysed the results.

All authors critically reviewed, improved and approved the manuscript.
